# A New Small-Bodied Azhdarchoid Pterosaur from the Lower Cretaceous of England and Its Implications for Pterosaur Anatomy, Diversity and Phylogeny

**DOI:** 10.1371/journal.pone.0058451

**Published:** 2013-03-18

**Authors:** Darren Naish, Martin Simpson, Gareth Dyke

**Affiliations:** 1 Ocean and Earth Sciences, University of Southampton, Southampton, United Kingdom; 2 Institute of Life Sciences, University of Southampton, Southampton, United Kingdom; Raymond M. Alf Museum of Paleontology, United States of America

## Abstract

**Background:**

Pterosaurs have been known from the Cretaceous sediments of the Isle of Wight (southern England, United Kingdom) since 1870. We describe the three-dimensional pelvic girdle and associated vertebrae of a small near-adult pterodactyloid from the Atherfield Clay Formation (lower Aptian, Lower Cretaceous). Despite acknowledged variation in the pterosaur pelvis, previous studies have not adequately sampled or incorporated pelvic characters into phylogenetic analyses.

**Methodology/Principal Findings:**

The new specimen represents the new taxon *Vectidraco daisymorrisae* gen. et sp. nov., diagnosed by the presence of a concavity posterodorsal to the acetabulum and the form of its postacetabular process on the ilium. Several characters suggest that *Vectidraco* belongs to Azhdarchoidea. We constructed a pelvis-only phylogenetic analysis to test whether the pterosaur pelvis carries a useful phylogenetic signal. Resolution in recovered trees was poor, but they approximately matched trees recovered from analyses of total evidence. We also added *Vectidraco* and our pelvic characters to an existing total-evidence matrix for pterosaurs. Both analyses recovered *Vectidraco* within Azhdarchoidea.

**Conclusions/Significance:**

The Lower Cretaceous strata of western Europe have yielded members of several pterosaur lineages, but Aptian pterosaurs from western Europe are rare. With a pelvis length of 40 mm, the new animal would have had a total length of c. 350 mm, and a wingspan of c. 750 mm. Barremian and Aptian pterodactyloids from western Europe show that small-bodied azhdarchoids lived alongside ornithocheirids and istiodactylids. This assemblage is similar in terms of which lineages are represented to the coeval beds of Liaoning, China; however, the number of species and specimens present at Liaoning is much higher. While the general phylogenetic composition of western European and Chinese communities appear to have been approximately similar, the differences may be due to different palaeoenvironmental and depositional settings. The western Europe pterodactyloid record may therefore be artificially low in diversity due to preservational factors.

## Introduction

Southern England is well known as a source of Cretaceous pterodactyloid pterosaur remains with some of the most taxonomically significant specimens coming from such strata as the Purbeck Limestone Group, Hastings Group and Wealden Group of the Wealden Supergroup, and Cambridge Greensand [1]. These include the holotype species and specimens of *Ornithocheirus* (*O. simus* (Owen, 1861)) [2], *Coloborhynchus* (*C. clavirostris* Owen, 1874) [3], *Istiodactylus* (*I. latidens* (Seeley, 1901)) [4] and *Lonchodectes* (*L. compressirostris* (Hooley, 1914)) [5].

The Cretaceous strata of the Isle of Wight are well known for their diverse dinosaurian assemblage [6], but both the Wessex and Vectis formations have also yielded pterosaurs [7]–[9]. *Istiodactylus latidens*, the first istiodactylid to be recognised, was originally named from the Vectis Formation [4] but may also be present in the Wessex Formation [9]; possible additional istiodactylid taxa, represented only by teeth, are also known from the Wessex Formation [8]. The Wessex Formation has also yielded the crested ornithocheirid *Caulkicephalus trimicrodon* [10], indeterminate azhdarchoid remains [11], and teeth suggested to belong to gnathosaurine ctenochasmatids and lonchodectids [8], [9]. A number of younger Cretaceous units on the Isle of Wight could potentially yield pterosaurs: on the mainland, pterosaur remains are known from the Chalk Formation, the most recently described of which are three cervical vertebrae from the Coniacian part of the unit [12], possibly referable to the azhdarchoid clade Tapejaridae (here used in the restrictive sense favoured by Martill and Naish [13]). Here, we report the partial pelvis and associated sacral and dorsal vertebrae from a small pterodactyloid that appears to represent a new taxon. The specimen was discovered in land-slip sediments on the Isle of Wight belonging to the Atherfield Clay Formation.

### Systematic Paleontology

Pterosauria Kaup, 1834.

Pterodactyloidea Plieninger, 1901.

Lophocratia Unwin, 2003.

Azhdarchoidea Nessov, 1984 (sensu Unwin, 1992 [14]).

*Vectidraco daisymorrisae*, Naish, Simpson, Dyke sp. nov. urn:lsid:zoobank.org:act:500375BE-D544-4539-B9ED-375AE25243C2.

#### Holotype specimen

**Figure 1:**
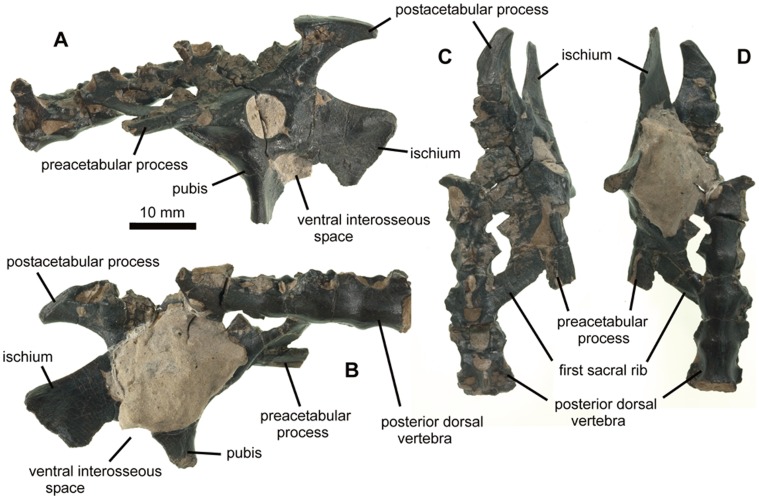
NHMUK PV R36621, holotype of *Vectidraco daisymorrisae* (except right ischium: see **Figure 2**). A, specimen as seen from left side, showing lateral surface of left side of pelvis and associated vertebrae; B, specimen as seen from right side, showing medial surface of left side of pelvis and associated vertebrae; C, specimen in dorsal view, anterior pointing down the page; D, specimen in ventral view, anterior pointing down the page.

**Figure 2:**
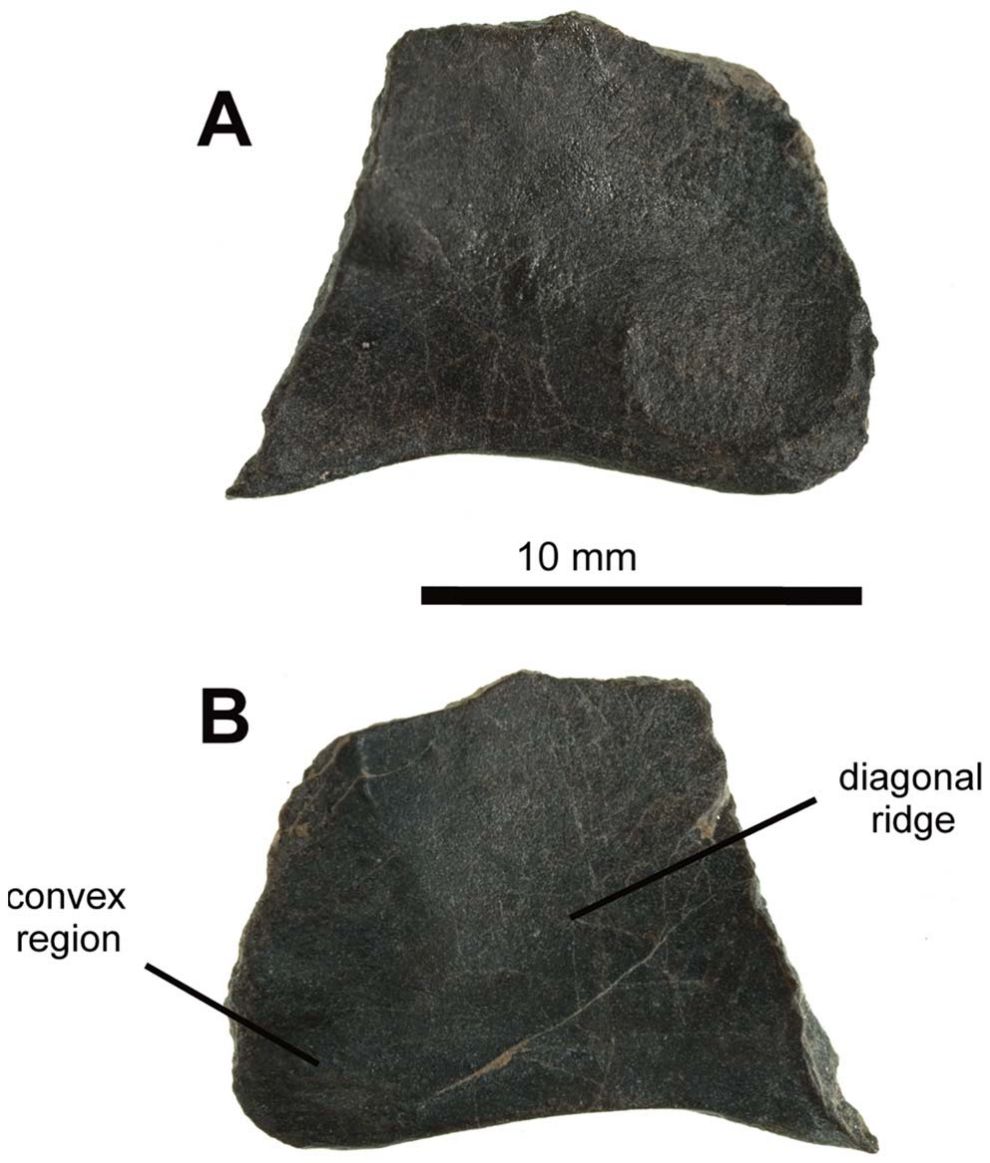
NHMUK PV R36621, incomplete right holotype ischium of *Vectidraco daisymorrisae*. A, medial view, showing largely featureless medial surface; B, lateral view, showing poorly expressed posteroventral convex region connected to feint diagonal ridge extending anterodorsally (compare with Figure 1A, 3).

**Figure 3:**
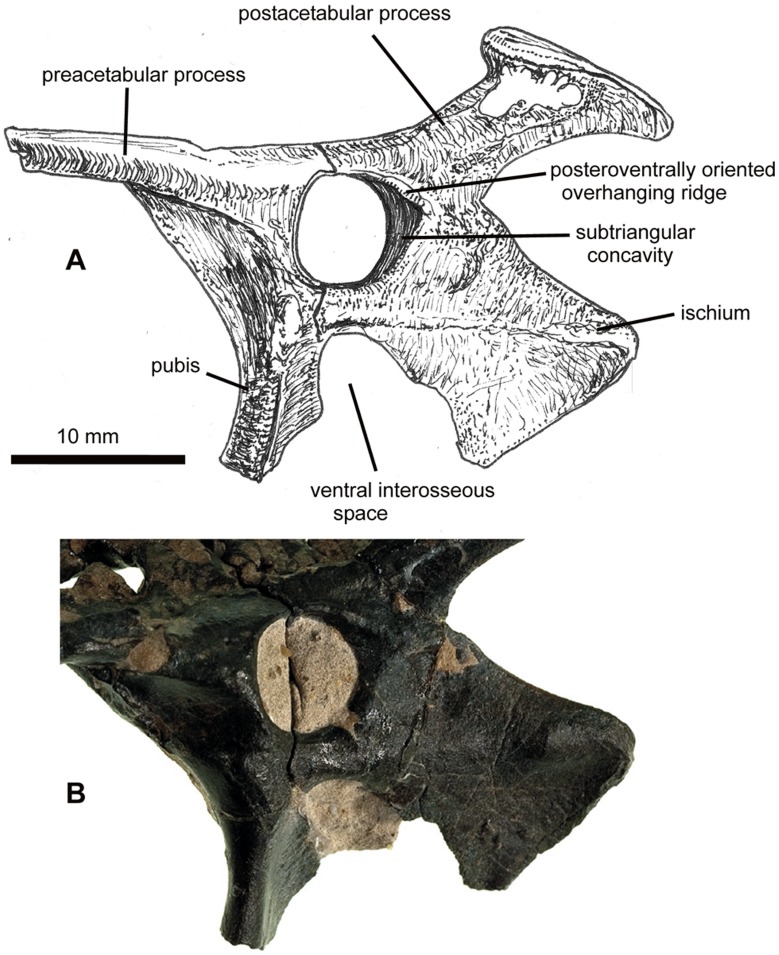
NHMUK PV R36621, left side of pelvis of *Vectidraco daisymorrisae*. A, diagrammatic representation, with associated vertebrae and matrix shown removed; B, detail of lower part of pelvis, showing autapomorphic subtriangular concavity and associated dorsal ridge posterior to acetabulum. The detailed anatomy of the ischium is more obvious on the left side than the right (compare with Figure 2).

**Figure 4:**
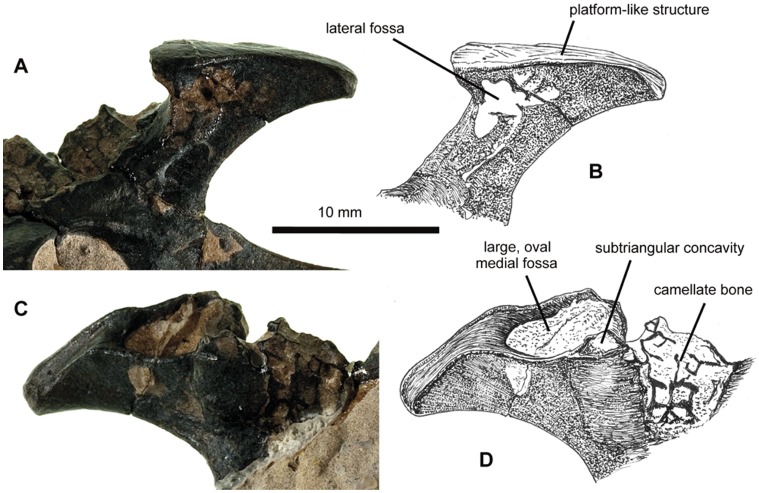
NHMUK PV R36621, left postacetabular process of *Vectidraco daisymorrisae*. A, postacetabular process in lateral view; B, diagrammatic interpretation of postacetabular process in lateral view, showing lateral fossa located anterodorsally and close to apex; note that apex is covered by a convex, platform-like structure that wraps around the apex posteriorly but is flatter anteriorly; C, postacetabular process in medial view; D, diagrammatic interpretation of postacetabular process in medial view showing large oval fossa and smaller, associated subtriangular cavity; anteroventral to both, damage to the bone surface reveals the camellate interior of the bone.

**Figure 5:**
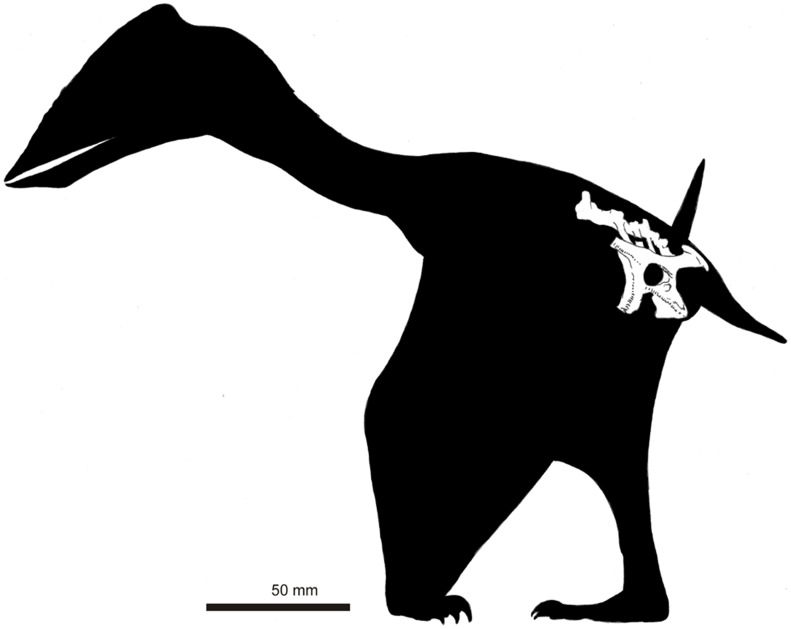
Speculative reconstruction of *Vectidraco daisymorrisae*. We assume that *Vectidraco* was similar in overall morphology and proportions to *Tapejara* and other small-bodied azhdarchoids, was edentulous, possibly crested, in possession of relatively short wings, and capable of parasagittal quadrupedal locomotion as shown here. Total length = c. 350 mm, wingspan = c. 750 mm.

NHMUK (Natural History Museum, London) PV R36621, partial pelvis and associated sacral vertebrae (Figure 1–5).

#### Etymology

*Vectis*, Greek, Isle of Wight and *draco*, Greek, dragon, hence ‘dragon from the Isle of Wight’. Specific name honors Daisy Morris, finder of the holotype.

#### Discovery, locality and age

The specimen was collected by the Morris family from a scree slope near the foot of the cliff immediately west of Atherfield Point: their attention was drawn to a collection of small bones which had weathered out of a block from the basal part of the marine Atherfield Clay Formation of the Lower Greensand Group. The matrix matches the silty clay of the Chale Clay Member [15], which is of Early Aptian, *Deshayesites forbesi* Zone, *Deshayesites fittoni* Subzone age [15]. The probable source horizon is several metres above the Perna Bed Member, itself characterised by a hard sandstone ledge which can be traced from beach level at Atherfield Point to the top of the cliff further west towards Shepherd’s Chine. The Chale Clay Member is characterised by the presence of selenite crystals, red clay ironstone nodules and uncommon ammonites of the genera *Deshayesites* and *Roloboceras*. Rare, isolated teeth of pterosaurs have also occasionally been found here (MIS, pers obs). This locality is traditionally referred to as ‘Atherfield’ in early literature, and is near the classic site known locally as ‘Tie Pits’, from which significant historical vertebrate remains have been recovered, notably the 1904 specimen of the goniopholidid crocodyliform *Anteophthalmosuchus hooleyi* [16], [17] and the 1914 specimen of the iguanodontian dinosaur *Mantellisaurus atherfieldensis* [18], [19]. Tie Pit ledge itself is an offshore reef-forming part of the Atherfield Bench immediately to the west of the Mexon Rocks. When first discovered, the specimen was a scattered assemblage of small phosphatised bones exposed on the surface of a weathered mass of Atherfield Clay. After a careful search of the immediate area, all of the elements were collected by the Morris family, along with a sample of the matrix. Later searches failed to locate any additional material. The exact site has since been washed away by the action of the sea. The only other rocks exposed at this site are the non-marine, bluish shales belonging to the Barremian-early Aptian Vectis Formation of the Wealden Group. However, the matrix and exact locality of the discovery precludes any attribution to that unit. A permit is not required for collection from the site.

#### Diagnosis

Pterodactyloid with subtriangular concavity posterodorsal to the acetabulum (Figure 1A, 3), overhung dorsally by a posteroventrally oriented ridge (Figure 1A, 3), and undivided, suboval fossa present on anteromedial surface of postacetabular process (Figure 1B, 4C–D), adjacent to convex dorsal surface. All three features represent autapomorphies of this taxon. The combined presence of a postacetabular process on the ilium where the shaft is elongate and where the entire length of the postacetabular process is similar in length to the convex terminus of the postacetabular process is also regarded as autapomorphic.

#### Nomenclatural acts

The electronic edition of this article conforms to the requirements of the amended International Code of Zoological Nomenclature, and hence the new names contained herein are available under that Code from the electronic edition of this article. This published work and the nomenclatural acts it contains have been registered in ZooBank, the online registration system for the ICZN. The ZooBank LSIDs (Life Science Identifiers) can be resolved and the associated information viewed through any standard web browser by appending the LSID to the prefix “http://zoobank.org/”. The LSID for this publication is: urn:lsid:zoobank.org:pub:1E226E5F-4020-4C8C-BD7C-09F4D0BD8E74. The electronic edition of this work was published in a journal with an ISSN, and has been archived and is available from the following digital repositories: PubMed Central, LOCKSS.

#### Description

The specimen includes most of the left side of the articulated pelvis (Figure 1): the prepubis, ventral part of the pubis and part of the anterior process of the ilium are missing, and adhering matrix (which cannot be removed without damaging the specimen) obscures the interior of the acetabulum and much of the medial surface of the pelvis. Much of the isolated right ischium was also discovered (Figure 2). The specimen is small, with a total preserved pelvis length of 40 mm and a maximum height of 27 mm (the pelvis would have been c. 20–30 mm longer when complete; the anterior part of the preacetabular process is missing). Closed sutures between all three pelvic bones, the fusion of three of the four preserved neural arches to their adjacent centra, the fusion of the sacral ribs to the ilium and to the neural arches, fusion between three of the four preserved centra and fully formed, fully ossified pelvic components show that the specimen was close to skeletal maturity, exhibiting an ontogenetic stage reported in other mature or near-mature pterosaur specimens [20], [21]. It therefore appears to have been a small adult, and not a juvenile of a larger-bodied taxon.

The preserved part of the preacetabular process is dorsoventrally compressed and wider than deep (Figure 1A, C–D). Its tip is missing. A sharp-edged lateral ridge extends along its lateral side: this becomes rounder posteriorly, eventually terminating at the acetabular border. It is possible that this structure corresponds to the insertion point for the ambiens muscle. Ventral to this structure, a subtle concavity takes up most of the space anterior to the acetabular border. There is no trace of an iliopubic suture. The acetabulum is sub-circular (7 mm long and 7 mm tall) (Figure 1A, 3): the acetabular rim projects further laterally at the anterior end than at the posterior end. Adjacent to the posterior border of the acetabulum, a shallow triangular notch is present on the lateral side of the ilium (Figure 3). This structure has not previously been reported in a pterosaur and is an autapomorphy of this new taxon. A shallow, sub-rounded concavity is present posteroventral to this notch (Figure 3). It is located just dorsal to the presumed position of the obliterated ilioischiadic suture.

The postacetabular process of the ilium projects posterodorsally as a prominent, superficially ‘T-shaped’ process, the apex or terminus of which is much higher dorsally than the dorsal edge of the rest of the ilium (Figure 1A, 3). In lateral view, the process has a narrow shaft with sharp, slightly concave dorsal and ventral margins (Figure 1A, 3–4). A gently convex, obliquely oriented platform (12 mm long) forms the apex (Figure 3–4). Prominent edges differentiate this apex from the rest of the shaft. At its posterior end, the surface descends ventrally to wrap around the posterodorsal part of the shaft (Figure 4). A series of connected depressions are located on the lateral surface of the shaft, ventral to the anterior part of the apex (Figure 4A–B). The medial surface is more complicated. A deep, oval concavity, its longest axis being the anteroposterior one, takes up most of the anterodorsal part of the apex; a small, subtriangular concavity is present anteromedial to the larger structure (Figure 4C–D). Anteroventral to both structures, the anteromedial surface of the postacetabular process is occupied by a honeycomb-like arrangement of bony walls (Figure 4C–D): this reveals the presence of pneumatic, camellate bone texture. Pneumatisation of the pterodactyloid ilium has been reported for the ornithocheirid *Anhanguera* [22]; its presence in *Vectidraco* suggests that it is more widespread in Pterodactyloidea, though whether it evolved independently in these taxa or is phylogenetically ubiquitous awaits data from other taxa. Camellate bone has long been known from other pterodactyloids and been illustrated in the rostrum [13] and vertebrae and other post-cranial elements [23] of several azhdarchoid taxa. *Vectidraco* demonstrates the presence of pneumatisation of the pterodactyloid postacetabular process.

The pubis is incomplete ventrally and posteriorly: it descends anteroventrally from the anterior part of the ilium and has a gently concave anterior margin (Figure 1, 3). This margin is thick and rounded. The more posterior part of the bone is a delicate lamina that is oriented such that its posterior border is located some distance medially relative to the anterior border (Figure 1A, 3). A similar dorsoventrally-aligned thickened anterior ridge and posterior lamina occur in *Gegepterus changae* from the Yixian Formation [24]. Breakage at the ventral end of the pubis reveals a tear-drop-like cross-sectional shape. The posterodorsal part of the pubis form part of the margin to a relatively large, oval foramen, ventral to the acetabulum (Figure 1A, 3). The anteroventral part of the ischium forms the opposite side of the foramen. The pubis and ischium did not, therefore, form a continuous plate like that seen in some pterodactyloids, but were separated by a ventral opening that could be referred to as a thyroid fenestra. However, since the structure in *Vectidraco* appears non-homologous with the thyroid fenestrae present in other diapsids [25], [26], we have opted to use the neutral term ‘ventral interosseous space’ from hereon.

The ischium is a parasagitally-aligned sheet of bone (Figure 1A–B, 3). Both ischia are preserved (the right ischium is incomplete dorsally and is the only part of the right side of the pelvis yet discovered (Figure 2)). The bone is approximately square in lateral view, with sub-parallel anterior and posterior margins, and a straight-edged ventral margin aligned at 90° to the posterior margin. The posterior margin is very slightly concave; the ventral and anterior borders are particularly thin (Figure 1A, 2). On the lateral side of the ischium, a subtle diagonal ridge extends from just beneath the acetabulum to the posteroventral ‘corner’ of the bone (Figure 1A, 2, 3). A low, gentle convexity occurs at the posteroventral termination of the ridge. Both the ridge and convexity are less obvious on the right ischium than the left: they appear indistinct in Figure 2.

Four vertebrae are preserved, the posterior three of which are connected to the ilium by dorsoventrally-flattened sacral ribs (Figure 1). These three are therefore interpreted as the anteriormost of the sacral vertebrae: the lateral parts of two additional sacral ribs project medially from the posterior part of the ilium (Figure 1A, C–D), so five sacrals were present in life. The most anterior preserved vertebrae does not possess elongate sacral ribs (Figure 1) and is identified as the posteriormost dorsal. The dorsal is the longest of the four vertebrae (10.5 mm long); the preserved sacrals are between 7 and 8.5 mm long. The centra decrease in height posteriorly. The ventral surfaces of the dorsal and first sacral centra are rounded while those of the second and third sacral centra are almost flat (Figure 1B). Ventral bulges on the dorsal centrum and first sacral look superficially like fully fused junctions between adjacent centra (Figure 1B) but are actually located close to the mid-lengths of these two vertebrae. On the lateral surfaces of all preserved centra, sub-oval pneumatic foramina occur posterior to the bases of the diapophyses (Figure 1A–B). The neural spine on the third sacral is missing while those on the remaining vertebrae lack their apices (Figure 1A–B); the tallest spine is that on the dorsal vertebra: this spine is subtriangular in anterior or posterior view and possesses two small, teardrop-shaped shallow fossae at the base of its anterior face. Prezygapophyses are best preserved on the first sacral vertebra and are anteroposteriorly short, dorsally convex and widely separated. The dorsal vertebra’s neural canal is slightly wider than it is tall (4 mm×3 mm). The anterior articular surface of the dorsal vertebra’s centrum is also wider than tall (6 mm×4 mm), but with details obscured by damage and matrix.

The sacral ribs project posterolaterally from the specimen’s left side (Figure 1C–D). The best preserved of them belongs to the first sacral vertebra; at its point of emergence from the vertebra, it extends from just posterior to the prezygapophysis to the base of the postzygapophysis (Figure 1C). It is similar in length to the adjacent centrum. The rib is marked by striations on its posterior part. Lateromedially elongate spaces are present between the three anterior sacral ribs, whereas the posterior ribs are in such close contact that the space is reduced to a slit (Figure 1A, C–D).

Estimating the total size of *Vectidraco* is difficult given that pterodactyloids are highly variable in relative body proportions and wing shape [27]. By comparing the *Vectidraco* holotype with a skeletal reconstructions of the small azhdarchoid *Tapejara* created by Ross Elgin [28], we estimate that *Vectidraco* would have had a total length (from the tip of the rostrum to the tail) of c. 350 mm and a wingspan of c. 750 mm, though it should be understood that these are approximate given the above caveats. While the size of a fully mature individual remains unknown, it is unlikely to have been much larger. Accordingly, *Vectidraco* probably represents the smallest azhdarchoid yet reported: other small azhdarchoids (the Liaoning tapejarids *Sinopterus* and *Huaxiapterus*) have wingspans of 1140 and 1430 mm respectively [27]. A speculative reconstruction (Figure 5) assumes that *Vectidraco* was, like better known azhdarchoids, a proficient quadruped with proportionally short wings and an edentulous, probably crested skull.

## Discussion

### Implications for Pterosaur Diversity

*Vectidraco* augments the known pterosaur diversity of Lower Cretaceous western Europe and helps fill an ‘early Aptian gap’ in the western European record. As indicated above, Lower Cretaceous western European pterosaurs might seem relatively diverse and well known, since they include the Valanginian taxa from the Hastings Group of southern England [9], the Barremian tapejarid, istiodactylid and ornithocheirid remains from Las Hoyas and Galve in Spain [29]–[31], the Barremian-early Aptian taxa of the Wealden Group in southern England [8]–[11], *Prejanopterus curvirostra* from the Aptian of La Rioja in Spain [32] and the Albian taxa from the Cambridge Greensand of England [33]. Among these taxa, only *Prejanopterus* from La Rioja and *Istiodactylus latidens* from the Vectis Formation of the Isle of Wight are from the early Aptian. Unwin et al. [34] referred to a jaw fragment from the Atherfield Clay Formation, referred to *Ornithocheirus*: aside from *Vectidraco* and the undescribed pterosaur teeth (presumed to be those of ornithocheirids on account of reported shape and size) noted here, this is the only reported Atherfield Clay Formation pterosaur. *Vectidraco* is thus significant in augmenting the poor western European record of Aptian pterosaurs.

However, pterosaurs can be considered well represented in Aptian strata elsewhere in the world: the partially Aptian [1], [34], [35] Yixian and Jiufotang formations, for example, have yielded high numbers of species representing most pterosaur lineages thought to exist during the Early Cretaceous. The Yixian Formation – dated to the late Barremian-early Aptian [1], [35] – preserves anurognathids (*Dendrorhynchoides curvidentatus*), ornithocheiroids (*Feilongus youngi*, *Haopterus cuiae* and others), ctenochasmatids (*Eosipterus yangi*, *Gegepterus changae* and others) and tapejarids, while the Jiufotang Formation – dated to the Aptian [1], [36] – has revealed istiodactylids, ornithocheirids, dsungaripteroids and tapejarids [1]. The Aptian-Albian Qingshan Formation of Shandong, China, has yielded possible dsungaripterid material [1] and the dsungaripterid *Lonchognathosaurus acutirostris* is from the ?Aptian-Albian Lianmuqin Formartion of Xinjiang Uygur Zizhiqu, China [37]. Ornithocheirid material (possibly belonging to *Coloborhynchus*) is known from the Aptian-Albian Züünbayan Formation of Dornogovi, Mongolia [38]. Other Eurasian Aptian strata yielding pterosaurs (mostly ornithocheirids) are known, including the Khilok and Ilek formations of Russia [1].

The pattern emerging from these comparisons is that the pterosaur assemblages of Barremian and Aptian western Europe were similar in terms of lineage representation, and hence community structure, to the far better-known assemblages of Liaoning, with small-bodied azhdarchoids (e.g. *Vectidraco*, *Europejara*) and small-bodied, slender-snouted pterodactyloids (e.g. *Prejanopterus*) occurring alongside larger-bodied istiodactylids and ornithocheirids. Even after the Aptian, there is evidence that small-bodied azhdarchoids persisted in western Europe: Martill et al. [39] reported three cervical vertebrae from the Coniacian Chalk Formation of Kent that are possibly referable to the azhdarchoid clade Tapejaridae (here used in the restrictive sense favoured by Martill and Naish [13]). If the western European Lower Cretaceous pterosaur assemblage is approximately similar to that of Liaoning in terms of the number of lineages represented, why is the number of species and specimens so much higher at Liaoning? We suggest that major differences in the taphonomic settings of the respective localities explains, in part, this phenomenon: the Yixian and Jiufotang formation are dominated by fine-grained lacustrine deposits, interspersed by volcanic sediments that resulted in mass death assemblages and the rapid burial of small vertebrate carcasses [40]. In contrast, western European deposits of coeval age (e.g. the sediments of the Wealden Supergroup) are typically dominated by floodplain and estuarine depositional settings where vertebrate remains were typically broken apart, scattered and scavenged before the more resistant parts were incorporated into the sediment record [41]. While the small pterosaurs and theropod dinosaurs discovered at Liaoning are often articulated and near-complete (e.g. [42]–[46]), those from the Wealden typically consist of isolated bones [47], [48]. In conclusion, the western European Lower Cretaceous pterosaur assemblage is most reasonably interpreted as strongly biased by the absence of sites where pterosaurs were both taxonomically diverse, and readily incorporated into the sediment record: a position that agrees with quantitative studies of pterosaur diversity across time [49]. We recognise, however, that environmental and other factors may also have contributed to differences in the respective pterosaur assemblages. Further work is needed to investigate this area.

### Phylogenetic Relationships

*Vectidraco daisymorrisae* can be unambiguously identified as a pterosaur on account of its combination of long pre-acetabular process, prominent and posterodorsally oriented, ‘T-shaped’ post-acetabular process, skeletal pneumatisation, and presence of (at least) five sacral vertebrae (Figure 1). While some of these characters (such as long preacetabular process and high number of sacral vertebrae) are present elsewhere within Reptilia, this combination is unique to Pterosauria.

**Figure 6:**
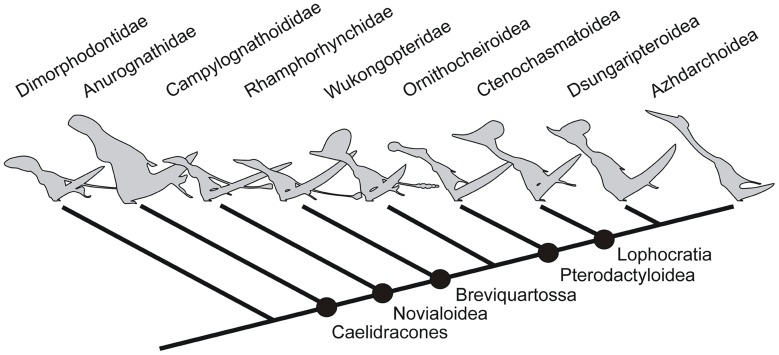
Highly simplified pterosaur phylogeny – topology based on that recovered by Unwin [**57**] – to show phylogenetic content of clade names used in the text. Caelidracones, Breviquartossa and Lophocratia are all node-based clades named by Unwin [57]. Novialoidea is a node-based clade named by Kellner [56]; it is essentially identical in content and definition to Unwin’s Longchognatha [57]. Pterodactyloidea was defined by Unwin [57] as the node-based *Pteranodon longiceps*+*Quetzalcoatlus northropi* clade but by Kellner [56] as the *Pterodactylus antiquus*+*Quetzalcoatlus northropi* clade. Neither definition is satisfactory since neither ‘captures’ all taxa in all topologies. Pterosaur silhouettes provided by Mark Witton.

Establishing the specimen’s affinities within Pterosauria is more difficult. While pterosaur workers have frequently published diagrams comparing and contrasting the pelves of different pterosaur taxa [21], [50]–[55], pelvic and/or sacral characters are mysteriously near-absent from phylogenetic studies of the group: neither Kellner [56] nor Unwin [57], for example, used a single pelvic character in their whole-pterosaur data sets of 74 and 60 characters, respectively, and pelvic and sacral characters are missing from the analyses of Kellner [58], Lü et al. [59] and Wang et al. [60]. Three pelvic characters were incorporated into the analyses of non-pterodactyloid pterosaurian data compiled by Unwin [61] and Andres et al. [62], and a single character was used by Andres and Ji [63] in their analysis of Pterodactyloidea. These characters concern the proportional length of the anterior iliac process (or preacetabular process), shape of the ischiopubic plate, and the form of the prepubis: they serve to differentiate the condition present in the most basal pterosaurs from the remaining members of the groups. The derived conditions (where the anterior iliac process is proportionally long, the ischiopubic plate is expanded anteroventrally and posteroventrally, and the ventral part of the prepubis is transversely expanded) are widespread in Unwin’s [57] proposed clade Caelidracones (defined by Unwin [57] as the node-based clade that includes *Anurognathus ammoni*, *Quetzalcoatlus northropi* and all descendants of their most recent common ancestor (Figure 6)). Ergo, these characters do not assist in identifying the affinities of *Vectidraco*, other than confirming its membership of Caelidracones. Within Caelidracones, *Vectidraco* differs from members of Anurognathidae in possessing a proportionally wide sacrum: its sacrum (measured as the distance between both ilia) is about twice as wide as the length of the ventral part of the ilium, whereas the ventral part of the ilium is approximately as long as the sacrum is wide in anurognathids [64]. The presence of a gap between the pubis and ischium also appears unique to Breviquartossa (the rhamphorhynchid+pterodactyloid clade (Figure 6)): the primitive condition for Pterosauria is to possess a continuous ischiopubic plate [61], with a ventral interosseous space between the two elements being seen only in Ornithocheiroidea (e.g., *Anhanguera piscator* [65], *Arthurdactylus conandoylei* [51]), Ctenochasmatoidea (*Pterodactylus antiquus* [66]) and Dsungaripteroidea (*Dsungaripterus weii* [67]). This character is known to be ontogenetically variable in some taxa: in juveniles of *Rhamphorhynchus*, the pubis and ischium are separate and only unite later during growth [68]. However, the ventral interosseous space is present in skeletally mature specimens of some taxa (e.g., *Dsungaripterus weii* [67]); it is conceivable that this is due to paedomorphosis.

Within Novialoidea (the node-based clade recognised by Kellner [56] that includes *Campylognathoides liasicus*, *Quetzalcoatlus northropi*, and all descendants of their most recent common ancestor: see Figure 6), the characters present within *Vectidraco* indicate membership of Pterodactyloidea. In contrast to non-pterodactyloids (including Wukongopteridae; e.g., *Darwinopterus linglongtaensis* [69]), the post-acetabular process of the ilium is strongly elevated and posterodorsally enlarged in *Vectidraco* (Figure 1A, 3–4). In non-pterodactyloids, the post-acetabular processes either lack a prominent posterodorsal enlargement or are approximately level with the dorsal surface of the rest of the ilium, or both. Some pterodactyloids possess the primitive state (e.g., *Germanodactylus rhamphastinus* [66]). Within Pterodactyloidea, ornithocheiroid pelves are quite different from *Vectidraco*: the postacetabular process of the ornithocheiroid ilium does not project well dorsal to the rest of the ilium, as it does in *Vectidraco*, nor does it possess a strongly narrowed shaft with a concave anterodorsal margin [21], [51], [52], [65]. In pteranodontids and nyctosaurids, the postacetabular processes are medially inclined such that their apexes contact, and fuse with, the neural spine lamina [70], [71].

*Vectidraco* is similar overall to *Pterodactylus*, regarded as a close relative of *Cycnorhamphus*, Ctenochasmatidae and differing combinations of other taxa by Kellner [56] and Unwin [57] and the studies based on these two works. Kellner [56] used Archaeopterodactyloidea for the node-based clade that includes *Pterodactylus*, *Ctenochasma* and kin, while Unwin [57] used Ctenochasmatoidea for the node-based clade containing *Cynorhamphus suevicus*, *Pterodaustro guinazui* and kin. *Pterodactylus* and *Vectridraco* share an anteriorly concave margin to the pubis, an elevated postacetabular processes with an expanded, posterodorsally convex apex, and an interosseous space [66]. However, in some character states (in *Pterodactylus*, the acetabulum is oval rather than circular, and the shaft of the postacetabular processes is proportionally shorter in *Pterodactylus* than that of *Vectidraco* [66]), *Pterodactylus* is less like *Vectidraco* than are members of Azhdarchoidea. This conflicting signal makes it difficult to determine from comparison alone whether *Vectidraco* and *Pterodactylus* might be closely related. Other putative relatives of *Pterodactylus* differ from *Vectidraco* in lacking a distinctly elevated, expanded apex to the postacetabular processes and an interosseous space [21], [50]. It appears most likely that the similarities shared by *Pterodactylus* and *Vectidraco* represent convergence: this is supported by the results of our cladistic analysis (see below).

At least some dsungaripterids (specifically *Dsungaripterus weii*) resemble *Vectidraco* in possessing an interosseous space [67]. In the unnamed Jurassic dsungaripterid DFMMh/FV (Dino-Park Münchehagen/Verein zur Förderung der niedersächsischen Paläontologie e.V.) 500 [53], however, a ventral interosseous space seems to be absent; furthermore, the ischiopubic plate is shallow compared to that of *Vectidraco* and most other pterodactyloids [53]. The Jurassic dsungaripterid DFMMh/FV 500 and *Dsungaripterus* also differ from *Vectidraco* in that the posterodorsal process is only slightly elevated relative to the rest of the ilium, the shaft is short, and the apex is poorly developed [53], [67]. These differences suggest that *Vectidraco* is not a member of Dsungaripteroidea.

Little information is available on the azhdarchoid pelvis: even in the many Liaoning Province tapejarid specimens this part of the skeleton is poorly preserved or highly incomplete, or both. However, *Vectidraco* resembles azhdarchoids such as *Tapejara* in possessing an elevated posterodorsal process on the ilium that has a narrowed shaft and a dorsally convex apex [72] (Figure 1A, 3–4); it is further similar to *Tapejara* in possessing fossae on the medial surface of the posterodorsal process (Figure 4C–D) (note that the postacetabular process of the ilium is wrongly labelled as the prepubis in Eck et al. [72]). Some azhdarchoid specimens reveal a complete ischiopubic plate and a posterodorsal process on the ilium that possesses a much shorter ‘shaft’ than *Vectidraco* [54]. The Crato Formation azhdarchoid specimen described by Sayão and Kellner [54] – regarded as a member of Neoazhdarchia by Unwin and Martill [73] – is similar in overall shape and the form of its posterodorsal process to the Santana Formation pelvis described by Bennett [74], suggesting referral of this latter specimen to Azhdarchoidea.

In conclusion, the presence of a strongly elevated, posterodorsally enlarged post-acetabular process of the ilium (Figure 1A–B, 3–4), and of a ventral interosseous space between the pubis and ischium (Figure 1A, 3), indicate referral of *Vectidraco* to Pterodactyloidea. The well developed and strongly elevated posterodorsal process with fossae on its medial side (Figure 1B, 4C–D) indicates that *Vectidraco* is most likely a member of Azhdarchoidea.

We were interested in testing this qualitative assessment further, and in seeing if any useful phylogenetic signal can be extracted from assessment of the character states present in the pterosaurian pelvis. We therefore compiled a list of morphological characters gleaned from examination of the pterosaur pelvis, including the prepubis and sacrum. Excluding autapomorphies, we found 23 characters that could be reliably coded for a phylogenetic analysis (see Text S1). It is peculiar that such a large amount of character information has been neglected in previous studies; Hyder et al. [21] also recently drew attention to the large amount of character information present within the pterosaur pelvis and showed how distinct and homogenous pelvic types could be matched with the clades recognised by some authors. The following characters were used in our analysis:-

#### 1. Terminal section of prepubis: anteroventral edge straight in dorsal view (0); convex in dorsal view (1)

**Figure 7:**
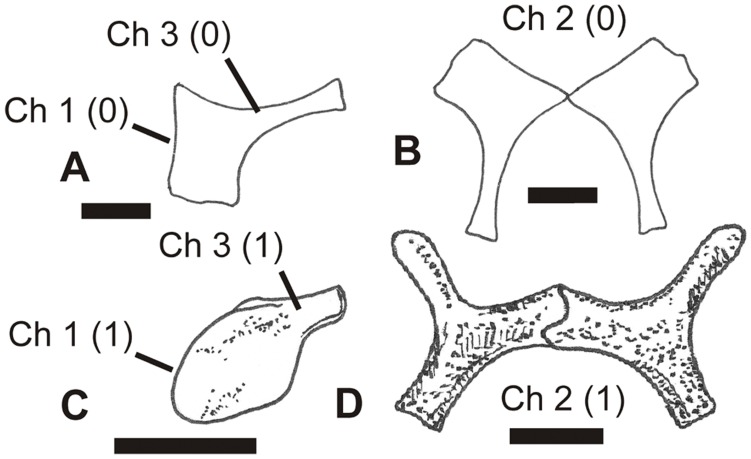
Different pterosaur prepubes in dorsal view, showing different states of characters 1–3, as illustrated by exemplar taxa. A, left half of prepubis of *Dimorphodon macronyx* in dorsal view, after Sayão and Kellner [50]; B, complete prepubis of *Dimorphodon macronyx* in dorsal view, after Sayão and Kellner [50]; C, left half of prepubis of *Eudimorphodon ranzii* in probable ventral view, after Wild [71]; D, complete prepubis of *Nyctosaurus gracilis*, after Williston [64]. See text for accompanying description of characters and their primitive (0) and derived (1) states. Scale bar = 10 mm.

The prepubis can be schematically likened to a ‘fan-shaped’ element that consists of a posterodorsally inclined ‘stem’ and an expanded anteroventral section (Figure 7). The form of the latter section is variable. Because the straight condition is apparently commoner in Jurassic pterosaurs [50], [54] (Figure 7A–B), we infer that it represents the primitive condition.

#### 2. Prepubis in ventral view: ‘paddle-shaped’ (0); with branching shape (1)

As described above, the prepubis includes an expanded anteroventral section connected to a posterodorsally inclined ‘stem’. In most pterosaurs, the anteroventral part is expanded and proportionally large. This is the case in *Dimorphodon* [54] (Figure 7A–B), *Eudimorphodon* [75] (Figure 7C), and the majority of pterodactyloids [50], [54]. A different condition is present in *Rhamphorhynchus* [76], *Nyctosaurus* [70] (Figure 7D) and *Pteranodon* [71]. Here, the anteroventral part of the prepubis is gracile, giving the whole bone a branching form very different from the paddle-like shape present in other taxa (Figure 7D). Because the gracile, branching prepubic shape is rare and phylogenetically restricted, it is assumed here to represent the derived condition.

#### 3. Shaft of prepubis: well differentiated from terminal section (0); short and poorly differentiated from terminal section (1)

Pterosaurs are variable with respect to the form of the prepubic shaft: it is proportionally long and well differentiated from the anteroventral part of the bone in some taxa (e.g., *Dimorphodon* [54] (Figure 7A–B), *Rhamphorhynchus* [76], *Pterodactylus* [66], *Gegepterus* [24]), but short and poorly differentiated from it in others (e.g., *Eudimorphodon* [75] (Figure 7C), *Pteranodon* [71]). We infer that the long-shafted condition is primitive since it is commoner and more widespread across phylogeny: the short-shafted condition seems to have arisen on a few, separate occasions.

#### 4. Ischiopubic plate: with pubis unexpanded (0); both elements expanded (1) (modified from Unwin [57])

**Figure 8:**
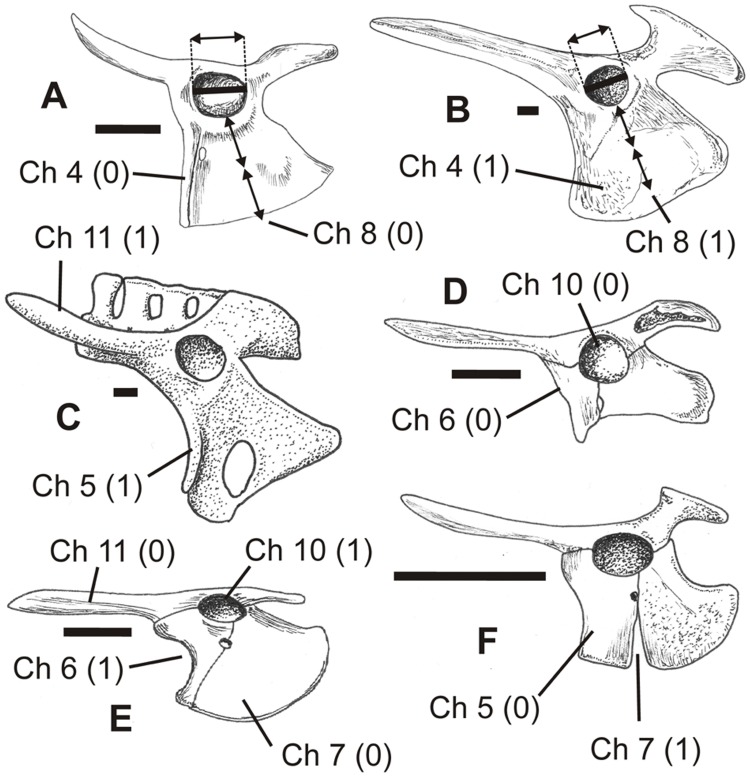
Pterosaur pelves in left lateral view showing different states of characters 4–8 and 10–11, as illustrated by exemplar taxa. A, *Dimorphodon macronyx*, after Wellnhofer [50]; B, Crato Formation neoazhdarchian MN 6588-V, after Sayão and Kellner [50]; C *Coloborhynchus spielbergi*, after Veldmeijer [48]; D, Kimmeridgian dsungaripterid DFMMH/FV500, after Fastnacht [53]; E, *Germanodactylus rhamphastinus*, after Wellnhofer [66]; F, *Pterodactylus antiquus*, after Wellnhofer [32]. See text for accompanying description of characters and their primitive (0) and derived (1) states. Scale bar = 10 mm.

In some non-pterodactyloid pterosaurs, the lateral surface of the pubis is anteroposteriorly short and almost rod-like (e.g., *Dimorphodon* [77]) (Figure 8A), while in other non-pterodactyloids (e.g., *Eudimorphodon* [75]) and in all pterodactyloids [21], [50]–[52] (Figure 8B–F) it is anteroposteriorly long and more similar in size to the ischium. The pubis is regarded here as ‘expanded’ if, in lateral view, it is more than half the length of the lateral surface of the ischium. Based on the prevalence of the ‘unexpanded’ pubis in non-pterodactyloids, it is here assumed that this condition is the primitive one. Unwin [61] used the character ‘ischiopubis: unexpanded (0), expanded (1)’ (character 48) and coded *Preondactylus*, *Dimorphodon* and *Peteinosaurus* as showing the primitive state; *Campylognathoides*, *Eudimorphodon*, *Scaphognathus*, *Sordes*, *Dorygnathus*, *Rhamphorhynchus* and Pterodactyloidea were coded with the derived state. In forms where the fusion of the pubis and ischium obscures their margins (e.g., *Nyctosaurus* [70]), the position of the obturator foramen provides an indication of the position of the puboischial symphysis and allows the size of the pubis to be estimated.

#### 5. Pubis: lateral surface essentially smooth (0); with anterolateral ridge for articulation with prepubis forming distinct separate section on bone (1)

The pubis in most pterosaurs is, viewed laterally, flattish, slightly convex or slightly concave (Figure 8F). In a number of taxa, however, a straight or anteriorly concave pillar- or vertical ridge-like structure forms the anterior margin to the pubis (e.g., *Campylognathoides* [78], *Coloborhynchus* [52], *Pteranodon* [71], *Tapejara* [72], *Vectidraco*) (Figure 8C). Its squared-off ventral end articulates with the prepubis. In those specimens where the symphysis between the pubis and ischium is obscured by fusion, this structure is still positioned further anteriorly than is the obturator foramen. This shows that at least part of the pubis still occurs posterior to the anterolateral ridge; in other words, that the anterolateral ridge is not synonymous with the pubic bone itself but, rather, is part of it. Because the absence of a vertical ridge or pillar is more widespread, especially in Triassic and Jurassic pterosaurs, we interpret its presence as the derived state.

#### 6. Pubis: with straight anterior margin (0); anterior margin concave (1)

The anterior margin of the pterosaur pelvis (as seen in lateral view) is straight or gently convex in some taxa (e.g., *Dimorphodon* [77] (Figure 8A), *Campylognathoides* [78], *Rhamphorhynchus* [76], *Pterodactylus* [66] (Figure 8F), *Gegepterus* [24]). However, in others, the same margin is concave, creating a ‘toe-like’ anteroventral extension for articulation with the prepubis. In some, the concavity is subtle (e.g., *Eudimorphodon* [75], *Anhanguera* [79], *Tapejara* [72]) and the anteroventral extension is tiny; in others, the concavity is prominent and the anteroventral extension is too (e.g., *Germanodactylus rhamphastinus* [66] (Figure 8E)). In forms with well developed concavity and anteroventral extension on the anterior pubic margin, the pubis mirrors the approximate form of the ischium. Because the straight condition seems to be more widespread in non-pterodactyloids it is assumed to represent the primitive one. An anteroventral expansion appears to be absent in the German dsungaripterid DFMMh/FV 500 (Figure 8D), and indeed Fastnacht [53] described the pubis as “rectangular in lateral view” (p. 276); however, the anteroventral part of the pubis is either missing or obscured in this specimen, so it is here coded ‘?’.

#### 7. Ischiopubic plate: continuous (0); ventrally open interosseous space between pubis and ischium (1)

In non-pterodactyloids, the ischiopubic plate is continuous, as is the primitive condition for diapsids. The plate is continuous in *Eudimorphodon* [75], *Dimorphodon* [77] (Figure 8A), *Rhamphorhynchus* [76], *Nyctosaurus* [70], *Pteranodon* [71] and *Germanodactylus* [66] (Figure 8E), for example. In contrast, a distinct notch or emargination, often resembling an inverted ‘V’, separates the ventral parts of the pubis and ischium in some taxa, including *Pterodactylus* [66] (Figure 8F) and *Vectidraco* (Figure 3). In at least some taxa, it appears that this character changed during ontogeny, with the ventral emargination of juveniles closing at maturity [68]. However, the emargination does appear to still be present in some taxa when they have reached maturity; *Rhamphorhynchus* was coded as polymorphic for this character because the subadults and adults we examined while coding [76] possessed both character states. The inclusion of the non-pterosaurs *Ornithosuchus* and *Herrerasaurus* in the Wang et al. data set used here [80] meant that we only examined the proximal, sheet-like portions of the pubis and ischium when coding for this character. In *Herrerasaurus*, both sections are deep and continuous ventral to the acetabulum [81]; in *Ornithosuchus*, an interosseous space is apparently present between the two elements [82].

#### 8. Ischiopubic plate: depth less than or equal to twice approximate length of acetabulum (0); depth more than twice length of acetabulum (1)

In non-pterodactyloid pterosaurs like *Dimorphodon* [77] (Figure 8A) and *Eudimorphodon* [75], the ischiopubic plate ventral to the acetabulum is relatively shallow, being less than twice as deep as the acetabulum is long. This condition is reversed in many other pterosaurs [21], [50]–[55] (Figure 8B).

#### 9. Ischia: ventrally separate (0); ventrally in contact (1)

**Figure 9:**
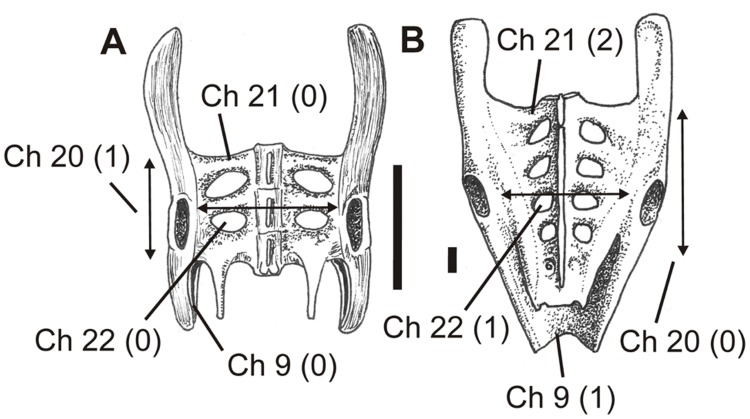
Pterosaur pelves in dorsal view showing different states of characters 9 and 20–22, as illustrated by exemplar taxa. A, *Rhamphorhynchus muensteri*, after Wellnhofer [32]; B, *Coloborhynchus spielbergi*, after Veldmeijer [48]. See text for accompanying description of characters and their primitive (0) and derived (1) states. Scale bar = 10 mm.

While little information is available on the three-dimensional form of the pterosaur pelvis, we know that the ischia are ventrally in contact in *Pteranodon longiceps* [71], *Coloborhynchus spielbergi* [52] (Figure 9B) and AMNH (American Museum of Natural History, New York) 22569 [74]. A lack of ventral contact appears typical for other pterosaurs (Figure 9A) and hence this is regarded as the primitive character state. In *Germanodactylus rhampastinus*, the pelvis is preserved in ventral view but partial collapse of the ischiopubic plates makes it difficult to determine whether an ischial symphysis was initially present [66]. This character is known to be sexually dimorphic in *Darwinopterus modularis* [83]; however, in other pterosaurs with sexually dimorphic pelves (e.g., *Pteranodon*), all individuals express the same character state [71].

#### 10. Acetabulum: circular or sub-circular (0); oval, longest axis horizontal (1) (Figure 8)

Most pterosaurs possess an approximately circular acetabulum (Figure 8D). However, a more oval-shaped acetabulum is present in *Pterodactylus antiquus* [66] (Figure 8F) and *Germanodactylus rhampastinus* [66] (Figure 8E).

#### 11. Preacetabular process of ilium: mostly straight along length (0); dorsally curved (1)

**Figure 10:**
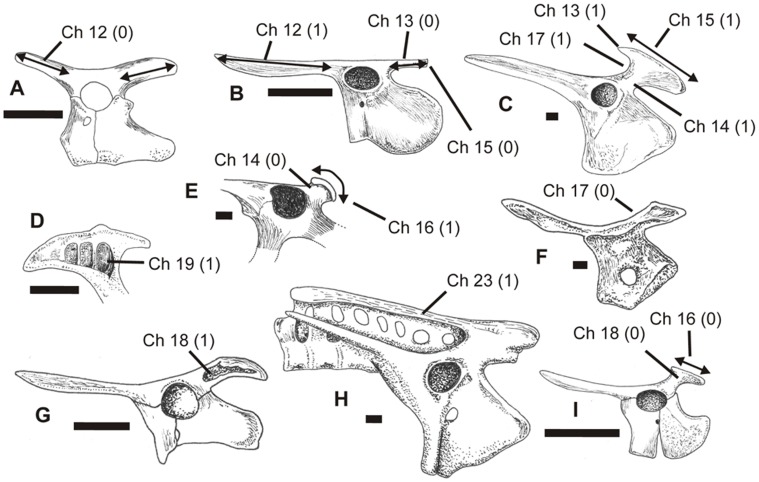
Pterosaur pelves in left lateral view (except D and F) showing different states of characters 12–19 and 23, as illustrated by exemplar taxa. A, *Eudimorphodon ranzii*, after Wild [71]; B, *Rhamphorhynchus muensteri*, after Wellnhofer [76]; C, Crato Formation neoazhdarchian MN 6588-V, after Sayão and Kellner [50]; D, postacetabular process in medial view of *Tapejara wellnhoferi*, after Eck et al. [72]; E, *Dsungaripterus weii*, after Young [67]; F, right side of pelvis in medial view of *Nyctosaurus gracilis*, after Williston [70]; G, Kimmeridgian dsungaripterid DFMMH/FV500, after Fastnacht [53]; H, *Pteranodon longiceps*, after Bennett [71]; I, *Pterodactylus antiquus*, after Wellnhofer [66]. See text for accompanying description of characters and their primitive (0) and derived (1) states. Scale bar = 10 mm.

In some pterosaurs (e.g. *Eudimorphodon ranzii* [75] (Figure 10A), *Germanodactylus rhampastinus* [66] (Figure 8E)), the preacetabular process is approximately straight as it projects from the anterodorsal part of the ilium. In others (e.g. *Pterodactylus antiquus* [66] (Figure 8F)) it curves dorsally. The straight condition is more common in Triassic and Jurassic pterosaurs and hence is hypothesised to be the primitive state.

#### 12. Preacetabular process of ilium: similar in length to postacetabular process (0); longer (1) (Unwin [61])

In Triassic pterosaurs (e.g. *Eudimorphodon ranzii* [75] (Figure 10A)), the pre- and postacetabular processes of the ilium are similar in length. In many pterodactyloids, the preacetabular process is longer [21], [50]–[55] (Figure 10B). Following Unwin [61], we assume that the short condition is the primitive one.

#### 13. Postacetabular process of ilium: with dorsal apex similar in height to rest of dorsal surface of ilium (0); strongly elevated relative to rest of ilium (1)

In non-pterodactyloid pterosaurs, the postacetabular process either continues posteriorly from the main body of the ilium such that its apex is similar in height to the part of the ilium dorsal to the acetabulum (e.g., *Eudimorphodon ranzii* [75] (Figure 10A)), *Rhamphorhynchus* [76] (Figure 10B)), or is elevated a short distance dorsal to it at its apex (*Dimorphodon macronyx*) [77] (Figure 8A). In some pterodactyloids (e.g. *Vectidraco* (Figure 1A, 3–4)), the postacetabular process projects at a high angle relative to the dorsal surface of the ilium [21], [50]–[55].

#### 14. Postacetabular process of ilium: no clear shaft prior to terminus (0); distinctly narrowed shaft region, much narrower than length of terminus (1)

In some pterosaurs, the postacetabular process projects posteriorly or posterodorsally with subparallel dorsal and ventral margins: there is either no distinct shaft region proximal to the terminus, or a very short, thick one. This condition is presumably primitive, since it is present in *Dimorphodon* [77] (Figure 8A), *Eudimorphodon* [75] (Figure 10A), *Rhamphorhynchus* [76] (Figure 10B), *Pteranodon* [71] (Figure 10H), *Anhanguera* and other ornithocheirid-like taxa [51]–[52], [79]. In contrast, a distinctly narrowed shaft region is present in a number of pterodactyloids, including *Pterodactylus* [66] (Figure 10I), AMNH 22569 [74], MN 6588-V [54] (Figure 10C), *Tapejara* [72] (Figure 10D) and *Vectidraco* (Figure 1A, 3–4). A distinctly narrowed shaft region of this sort is present across Azhdarchoidea, since AMNH 22569, MN (Museu Nacional, Rio de Janeiro) 6588-V and *Tapejara* are all hypothesised members of that clade [21]; the presence of this narrowed shaft in *Vectidraco* (Figure 1A, 3–4) indicates an azhdarchoid position for this taxon. Note that taxa in which there is no distinct terminus to the postacetabular process (e.g. *Eudimorphodon* [75] (Figure 10A)) are coded as possessing the short-shafted condition; this is because the entire postacetabular process is short in these taxa.

#### 15. Postacetabular process of ilium: terminus unexpanded, or less than length of acetabulum (0); terminus expanded, as long as or longer than acetabulum (1)

In Triassic and Early Jurassic pterosaurs, the postacetabular process either lacks an expanded terminus altogether [75] (Figure 10A–B), or has a small expansion that does not exceed the acetabulum in length [77] (Figure 10B). A much enlarged terminus is present in some pterodactyloid taxa [54], [72] (Figure 10C) and is interpreted as the derived condition.

#### 16. Postacetabular process of ilium: terminus with subhorizontal dorsal edge (0); terminus with convex dorsal edge (1)

In pterodactyloids with an elevated postacetabular process, the dorsoposterior margin may be either straight or convex when the pelvis is viewed laterally. The straight condition is present in *Pterodactylus* [66] (Figure 10I), *Cycnorhamphus* [21], *Nyctosaurus* [70] and *Pteranodon* [71] (Figure 10H) while the convex condition is present in dsungaripterids (*Dsungaripterus weii* [67] (Figure 10E) and DFMMh/FV 500 [53] (Figure 10G)), *Vectidraco* (Figure 1A, 3–4) and azhdarchoids [54], [72], [74] (Figure 10D).

#### 17. Anterodorsal edge of postacetabular process of ilium: straight or convex (0); concave (1)

In some pterodactyloids with an elevated postacetabular process, the anterodorsal edge of the process is straight or slightly convex: this is the condition that is more widespread in Triassic and Jurassic pterosaurs [75] (Figure 10A–B) and it is hence assumed to be primitive (Figure 10F). In some pterodactyloids, the anterodorsal edge is concave [54], [72] (Figure 10C). This character is not correlated with the presence of an expanded apex on the postacetabular process, since taxa with a large apex can still have a straight anterior edge to the process (e.g. *Coloborhynchus spielbergi* [52]).

#### 18. Lateral surface of postacetabular process of ilium: smooth (0); with fossae proximal to apex (1)

Several pterodactyloids possess fossae on the lateral surface of the postacebular process (e.g., DFMMh/FV 500 [53] (Figure 10G), *Vectidraco* (Figure 1A, 3A–B). The general absence of this character elsewhere in Pterosauria indicates that its presence is the derived state.

#### 19. Medial surface of postacetabular process of ilium: smooth (0); with fossae proximal to apex (1)

In some pterodactyloids (e.g., *Tapejara* [72] (Figure 10D), *Vectidraco* (Figure 4C–D)), fossae of the sort described in character 18 are also present on the medial surface of the postacetabular process. *Vectidraco* is unique in possessing a single, large, suboval fossa (Figure 4C–D), but fossae divided by vertical partitions are present in *Tapejara* [72] (Figure 10D).

#### 20. Width of sacrum: less than length of body of ilium (0); more than length of body of ilium (1)

In several pterosaurs (such as *Campylognathoides liasicus* [78], *Pteranodon* [71], AMNH 22569 [74] and *Vectidraco*), the maximum width of the sacrum (as measured from the lateral ends of both the left and right sacral ribs, between the mid-length points of the acetabula) exceeds the length of the ilium’s body (Figure 9A) (that is, measured from points dorsal to the subvertical anterior and posterior edges of the pubis and ischium, and not including the often long pre- and postacetabular processes (Figure 9)). Because a proportionally narrow sacrum (Figure 9B) is inferred to be primitive for Pterosauria (due to the presence of this narrow condition in *Anurognathus* [64]), the wide condition is interpreted as the derived one.

#### 21. Number of vertebrae incorporated into sacrum: three (or less) (0), four (1), five (2), six or more (3)

Pterosaurs are variable with respect to the number of sacral vertebrae; we assume here that pterosaurs ancestrally possessed a short sacrum of just three sacral vertebrae (Figure 9A), though this cannot be established with certainty and is the subject of current investigation (DWE Hone, pers. comm.). During evolution an increasing number of vertebrae became fused with the true sacrals (Figure 9B), such that some taxa (e.g., AMNH 22569 [74]) have a synsacrum incorporating as many as nine vertebrae. Because the non-pterosaurs *Ornithosuchus*, *Herrerasaurus* and *Scleromochlus* are coded here following their inclusion in the Wang et al. dataset [80], taxa with less than three sacral vertebrae are coded as possessing the primitive character state.

#### 22. Sacral ribs: separate (0); fused for part of their length, forming fenestrated sacral shield (1)

In many pterosaurs, the sacral ribs are coalesced for part of their length, forming a fenestrated sacral shield (Figure 9B). However, in *Eudimorphodon* [75] and *Campylognathoides* [78] the ribs are not coalesced and appear more like separate struts (Figure 9A). The presence of separate sacral ribs is assumed to be the primitive condition given its presence elsewhere in diapsids. However, unfused sacral ribs may also be present due to the ontogenetic stage of the individual, and it is also conceivable that paedomorphic taxa may have less ossification in the sacrum than other taxa.

#### 23. Neural spines: separate (0); fused to form supraneural plate (1)

Ancestrally, the sacral neural spines of pterosaurs are separate, and this is the condition in *Rhamphorhynchus* [76] (Figure 9A) and other non-pterodactyloids as well as *Pterodactylus* [66], *Germanodactylus* [66], *Anhanguera* [79], *Nyctosaurus* [70], *Tapejara* [72] and *Vectidraco* (Figure 1A). The spines are, however, partially fused through coalescence of the interspinous ligaments in *Coloborhynchus spielbergi* [52] (Figure 9B), *Pteranodon* [71] (Figure 10H), *Dsungaripterus weii* [67], the dsungaripterid DFMMh/FV 500 [53] and the neoazhdarchian MN 6588-V [54]. As with character 22, it is conceivable that this character varies with ontogeny.

## Methods

We hoped to see whether an analysis of pelvis-only characters (including characters of the prepubis and sacrum) would recover a tree or trees at all similar to those recovered from examination of a more representative sampling of characters across the skeleton. Accordingly, we ran analyses for two overlapping data sets. Our first analysis was based only on the 23 pelvic characters described above, coded for a representative selection of pterosaurs in which these characters are wholly or partly known (see Text S1). For our second analysis, we chose a recently published, comprehensive analysis of Pterosauria (we employed the analysis published by Wang et al. [80], only because it is recent as of the time of our study) and added our 23 pelvic characters before re-running (see Text S2: of the 129 codings for each taxon, the initial 106 are from Wang et al.’s study; codings 107–129 refer to our novel 23 pelvis characters). There was no overlap in character distribution between our 23 new characters and Wang et al.’s analysis [80] since, as is typical of pterosaur analyses (see above), their character set did not include a single pelvic character.

We note that the small size of the pelvic-only analysis (23 characters, 24 OTUs) is problematic and that such a low number of characters is not ideal considering the number of OTUs. The analysis should therefore be considered preliminary and we hope to expand on the data set used here in future.

Both data sets were subjected to parsimony analysis with equal weighting of characters using the phylogenetic program TNT [84]. Heuristic, unconstrained searches for optimal trees were conducted using 1000 replications (random addition sequence of taxa followed by TBR branch swapping). Character weights are not given “a priori” but are assigned during tree search, and thus, weighting depend only on the homoplasy inherent to the characters themselves [85]. For our pelvic-only character codings, an all-zero state outgroup was used; the Wang et al. analysis [80] used *Ornithosuchus longidens*, *Herrerasaurus ischigualastensis* and *Scleromochlus taylori* as outgroups. Characters present in members of the ingroup but wholly absent in the outgroup taxa (e.g., characters 17–19) were coded as ‘?’ (see Text S2).

## Results

**Figure 11:**
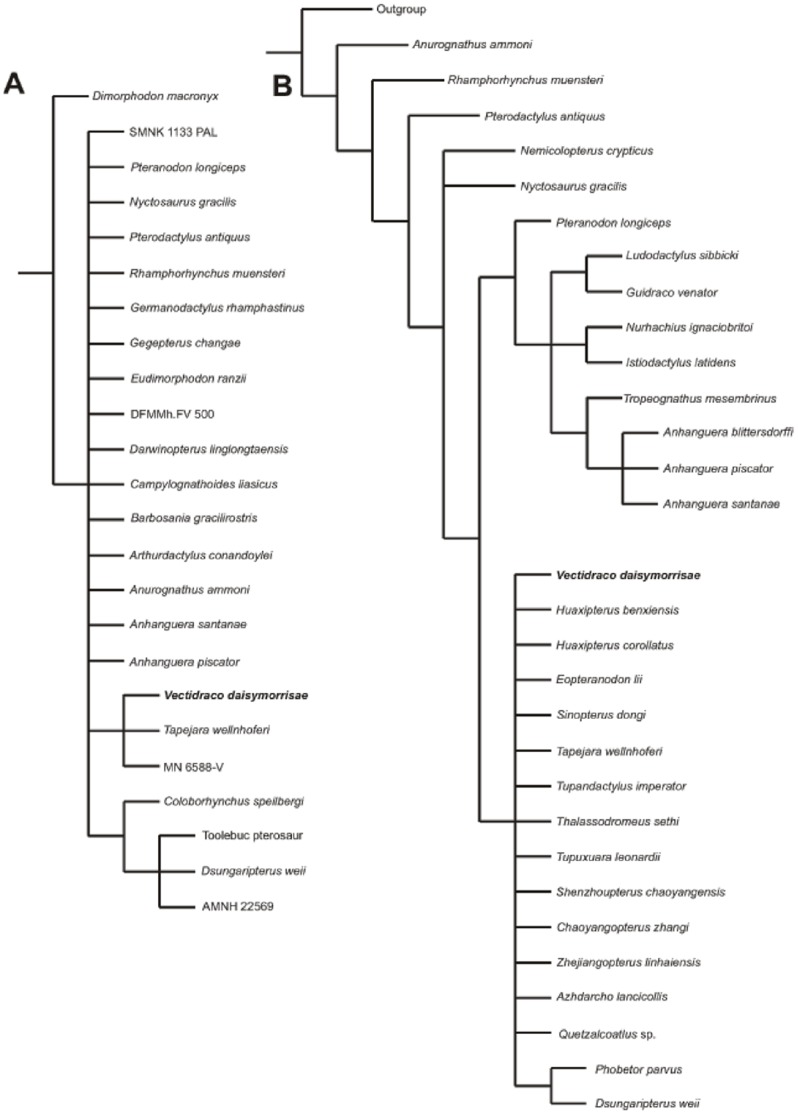
Trees resulting from inclusion of *Vectidraco* in both pelvis-only analysis and total-evidence analyses of Pterosauria. A, strict consensus of 3 MPTs (each 63 steps in length) resulting from analysis of the pelvis-only data set (with default outgroup); while little resolution is evident, *Vectidraco* groups with the azhdarchoids *Tapejara* and the Crato Formation neoazhdarchian MN 6588-V; B, Strict consensus tree of 12 MPTs resulting from analysis of the recoded Wang et al. [68] data set. The tree is illustrated here with a single outgroup, but *Ornithosuchus*, *Herrerasaurus* and *Scleromochlus* were all variously employed to root the analysis (following Wang et al. [68]): this did not result in any changes to resultant tree topologies. *Vectidraco* is recovered as part of a mostly unresolved clade containing azhdarchoids and dsungaripterids.

As expected for a small analysis consisting of near-equal numbers of characters and OTUs, our pelvic-only analysis recovered a poorly resolved strict consensus tree (Figure 11A). Nevertheless, the tree’s approximate structure is consistent with our general understanding of pterosaurian phylogeny: *Dimorphodon* was recovered as the sister-taxon to remaining pterosaurs, some pterodactyloids (including *Coloborhynchus*, *Dsungaripterus* and AMNH 22569) grouped together, and *Vectidraco*, *Tapejara* and MN 6588-V formed a clade (Figure 11A). This latter group represent Azhdarchoidea and the grouping of *Vectidraco* with *Tapejara* (a tapejarid) and MN 6588-V (a neoazhdarchian) supports the hypothesis that *Vectidraco* is a non-neoazhdarchian azhdarchoid. While the analysis is preliminary and limited, we conclude from these results that there is indeed a reliable phylogenetic signal in the pterosaur pelvis, and that at least some pterosaurian clades can be recovered on the basis of pelvic characters.

In the combined analysis (our 23 pelvic characters added to Wang et al.’s [68] set of characters from across the skeleton), the strict consensus recovered a topology where *Rhamphorhynchus* and *Anurognathus* represented successively more distant outgroups to Pterodactyloidea. Within the latter clade, *Pterodactylus* was the sister-group to the remainder; *Nemicolopterus*, *Nyctosaurus* and a clade containing all remaining pterodactyloids formed an unresolved trichotomy (Figure 11B). This ‘remaining pterodactyloids’ clade consisted of a *Pteranodon*+*Anhanguera* clade (containing *Istiodactylus* and all other pteranodontid-like and ornithocheirid-like taxa) and an unresolved dsungaripterid+azhdarchoid clade. *Vectidraco* belonged to this last clade (Figure 11B), again consistent with the hypothesis that it is a member of Azhdarchoidea. A 50% majority rule consensus tree resulted in a better resolved topology, with Azhdarchoidea consisting of distinct clades corresponding to Chaoyangopteridae, Dsungaripteridae, Azhdarchidae and a Thalassodromidae+Tapejaridae clade. All four clades formed a polytomy, however, and the lack of structure means that the close association of Thalassodromidae and Tapejaridae should not necessarily be considered strong support for the existence of this clade: the competing hypothesis is that Thalassodromidae is more closely related to Azhdarchidae, the two forming the azhdarchoid clade Neoazhdarchia [13], [57], [59]. While we feel that this topology better reflects the probable phylogenetic structure of Azhdarchoidea (the inclusion of Dsungaripteridae within Azhdarchoidea, however, is likely to be erroneous), we appreciate that equally parsimonious trees may produce different topologies and hence have chosen not to illustrate this majority rule consensus tree.

## Supporting Information

Text S1 **Character codings for pelvis-only analysis.**
(DOC)

Text S2 **Character codings for Wang et al. (2012) dataset, with the last 23 codings (characters 107–129) referring to the novel characters described in our text.**
(DOC)
