# Supplementary material for: A New Small-Bodied Azhdarchoid Pterosaur from the Lower Cretaceous of England and Its Implications for Pterosaur Anatomy, Diversity and Phylogeny
Source: PLoS One. 2013 Mar 18;8(3):e58451. doi: 10.1371/journal.pone.0058451 (PMC3601094; doi:10.1371/journal.pone.0058451)
Supplement: Text S1 — Character codings for pelvis-only analysis. (DOC) [file pone.0058451.s001.doc]

**Text S1. Character codings for pelvis-only analysis**

AMNH 22569 ???1010010 ??11111001 31?

*Anhanguera piscator* ???1111110 ??10000000 210

*Anhanguera santanae*  ???11111?0 1110000000 210

*Anurognathus ammoni* ?????????? ?1???????0 2??

*Arthurdactylus conandoylei* ???1??110? ??100000?1 30?

*Barbosania gracilirostris* ???1?110?? ???0?000?? ???

*Campylognathoides liasicus* ???1100100 0100000001 200

*Coloborhynchus speilbergi* ???1111110 1110110000 311

*Darwinopterus linglongtaensis* ?0?10111?? 01000000?1 210

DFMMh/FV 500 dsungaripterid ????0?0000 01100101?1 211

*Dimorphodon macronyx* 0000?000?0 10000000?? 110

*Dsungaripterus weii* ???1?11??0 ??10111000 311

*Eudimorphodon ranzii* 10110100?0 00000000?1 100

*Gegepterus changae* 1001?000?1 01000000?? ???

*Germanodactylus rhamphastinus* 10010101?1 01000000?0 2??

*Rhamphorhynchus muensteri*  010100[0/1]000 0100000001 000

MN 6588-V ?011??01?0 01111110?? 2?1

*Pterodactylus antiquus* 1001011001 0111101000 010

*Nyctosaurus gracilis* ?111?1011? 1110000001 310

*Pteranodon longiceps* 0111110110 0110100001 311

*Tapejara wellnhoferi* ???11101?? 011111101? ???

Toolebuc pterosaur ???11100?0 1110111??0 ???

*Tropeognathus* cf. *robustus* SMNK 1133 PAL ???111?0?0 ??10?000?? ???

*Vectidraco daisymorrisae* ???1111100 0?11111111 310
