# Supplementary material for: A New Small-Bodied Azhdarchoid Pterosaur from the Lower Cretaceous of England and Its Implications for Pterosaur Anatomy, Diversity and Phylogeny
Source: PLoS One. 2013 Mar 18;8(3):e58451. doi: 10.1371/journal.pone.0058451 (PMC3601094; doi:10.1371/journal.pone.0058451)
Supplement: Text S2 — Character codings for Wang et al. (2012) dataset, with the last 23 codings (characters 107–129) referring to the novel characters described in our text. (DOC) [file pone.0058451.s002.doc]

**Text S2. Character codings for Wang et al. (2012) dataset, with the last 23 codings (characters 107-129) referring to the novel characters described in our text.**

*Ornithosuchus longidens*

0000000?00000000???00000????000000??0?0000000000000000??00000000000000000000000000000000000000000000000000???0001010?0000????0000

*Herrerasaurus ischigualastensis*

0000000?00000000???00000????000000??0?0000000000000000??00000000000000000000000000000000000000000000000020???000001000000????0000

*Scleromochlus taylori*

000?0?0??0?000?0???00000?????????0????000?????0????0?0??00?0?0??0??00??000????00????00??0???0?0??????0??????????????????0?????000

*Anurognathus ammoni*

010001???0?????0???000?0????0??0?0??0?????????0???0000??0000110?0??0???100??0??????011??10000?1011????001????????????1???????02??

*Rhamphorhynchus muensteri*

0010100?00000010???00000????000000??0?0110000001101010??0000100000000000100000100011110020011?1012222210120101000/10000100000001000

*Pterodactylus antiquus*

0010101000000010???000110010001000??0?121?0?000???1000??0000120000011101000000100012110050021?10212222202010010010010110101000010

*Nyctosaurus gracilis*

00101010000000?0???00010????001000??0?0?110?0001211000??3000100111001001000000100013111030?3122222222?20???111?1011?1000000001310

*Nemicolopterus crypticus*

00101010000000?0???000111100011000??0?0?2?????0???1000??3000100?0?001??100000???0???11?15??2????21?22?2120???????????1????????31?

*Pteranodon longiceps*

1020101010300010???0001112?000110122110121010001211000??3000100111101011011101200012111041131222222223202001111101100110100001311

*Istiodactylus latidens*

00101011002???10???00?11110?0121?0??0?012?01?00??10000??2000101?1110?01???111020002?1?01411?22???????????????????????????????????

*Nurhachius ignaciobritoi*

0010101100?000?0???0001?????01110??????12?????0????000??200010101??0101???1?10200?22110?41?222?1212???21?????????????????????????

*Tropeognathus mesembrinus*

00101010003000110200101?????00110100100121010201211001010000100??????????????????????????????????????????????????????????????????

*Anhanguera santanae*

00101010103000110200101110010011010010012101010121100??10010100101101011??21102100????01411?22?1?????????????11111?01110000000210

*Anhanguera blittersdorffi*

00101010103000110200101?????00110100100121010101211001010010100??????????????????????????????????????????????????????????????????

*Anhanguera piscator*

001010101030001102001011100100110100100121010?012110010100101001011010110121112100221101411222?1??????2120???1111110??10000001210

*Ludodactylus sibbicki*

0010101000000010???0?01110010001011?1101110?0?0???1000??0010100??????????????????????????????????????????????????????????????????

*Guidraco venator*

0020101010300010???0?01?????000101151101110?0?0???1000??0000100??1101????????????????????????????????????????????????????????????

*Dsungaripterus weii*

201010100011011111000110????00100121110121111101211000??11011001111010010?000???0?2210??5?0311?121222?2120???1?11??0??10111000311

*Phobetor parvus*

001010100011011111000110????0010012111012?1?1?0???1000??1101100??????0???????????????????????1???????????????????????????????????

*Quetzalcoatlus* sp

00101010003010?14?000010????00100??????121???001??1000??300010011102122?0?00002010?20?10520311??21322?2?20???????????????????????

*Azhdarcho lancicollis*

??????????3????????????????????0????????????????????????300010?11102122???????????????105?0??????????????????????????????????????

*Zhejiangopterus linhaiensis*

00101010003010?0???00010????001000??0?012?1???0????000??300010011??2122?0?000???10?200??5??31?2?213???2??????????????1???????011?

*Chaoyangopterus zhangi*

1010101????0???????000????????????????????????0???1000??3000100?0101100???000???00?200?????31?2?21222321?0101????????????????????

*Shenzhoupterus chaoyangensis*

1010101101301020???0001?????0?20012411012?1???0???1000??3000100?01011?????0??0??0??200?????31???21222321?????????????????????????

*Tupuxuara leonardii*

00101011003010213400001?????0110012311012111130121100112300010011110100???00002001221010520311?1213???2??????????????????????????

*Thalassodromeus sethi*

00101011003010213400001112?001100123110121111301211000??3000100??????????????????????????????????????????????????????????????????

*Tupandactylus imperator*

00111011003010213301001??????110012211012?1??????????1133000100??????????????????????????????????????????????????????????????????

*Tapejara wellnhoferi*

001110110030102133000011111011100122110120111011211101133000100?0110100?0?00002001?210115203112?21????2120???11101??0110­11101????

*Sinopterus dongi*

00111011003010?13300001110001110112111012?1???1????101143000100?0101100???0000200??2111?52?31?2?21222321?????????????????????????

*Eopteranodon lii*

001110110??????1330000???????11??????????1???0????1?01143000000??1????0?????0??????211??5??31?2?21222?2??????????????????????????

*Huaxiapterus corollatus*

0011101????????1?310001????????????????????????????101143000100?01011?????000???0??211??5??3????21322321?????????????????????????

*Huaxiapterus benxiensis*

0011101100?0???13310001??????110?12111012?1???1???1101143000100?01011??????????????2???????3??2?21222?2120???????????????????????
